# Time-resolved infrared spectroscopic techniques as applied to channelrhodopsin

**DOI:** 10.3389/fmolb.2015.00038

**Published:** 2015-07-07

**Authors:** Eglof Ritter, Ljiljana Puskar, Franz J. Bartl, Emad F. Aziz, Peter Hegemann, Ulrich Schade

**Affiliations:** ^1^Experimentelle Biophysik, Institut für Biologie, Humboldt-Universität zu BerlinBerlin, Germany; ^2^Methods for Material Development, Helmholtz-Zentrum für Materialien und Energie GmbHBerlin, Germany; ^3^Institut für medizinische Physik und Biophysik, Charité – Universitätsmedizin BerlinBerlin, Germany; ^4^Fachbereich Physik, Freie Universität BerlinBerlin, Germany

**Keywords:** infrared spectroscopy, time-resolved spectroscopy, FTIR, IR-spectrometer, retinal proteins, channelrhodopsin

## Abstract

Among optogenetic tools, channelrhodopsins, the light gated ion channels of the plasma membrane from green algae, play the most important role. Properties like channel selectivity, timing parameters or color can be influenced by the exchange of selected amino acids. Although widely used, in the field of neurosciences for example, there is still little known about their photocycles and the mechanism of ion channel gating and conductance. One of the preferred methods for these studies is infrared spectroscopy since it allows observation of proteins and their function at a molecular level and in near-native environment. The absorption of a photon in channelrhodopsin leads to retinal isomerization within femtoseconds, the conductive states are reached in the microsecond time scale and the return into the fully dark-adapted state may take more than minutes. To be able to cover all these time regimes, a range of different spectroscopical approaches are necessary. This mini-review focuses on time-resolved applications of the infrared technique to study channelrhodopsins and other light triggered proteins. We will discuss the approaches with respect to their suitability to the investigation of channelrhodopsin and related proteins.

## Introduction

Marked with the first description of channelrhodopsin as a light-gated ion channel in 2002 (Nagel et al., 2002) the new field of optogenetics emerged and has since gone through rapid development. It utilizes light sensitive proteins like channelrhodopsins, bacteriorhodopsin, rhodopsin, blue light receptors (BLUF) (Kennis and Mathes, 2013), phytochromes (Yang et al., 2013), or engineered proteins (Möglich and Moffat, 2010) as tools to control some defined events in living cells by light (Zhang et al., 2006).

The most commonly used channelrhodopsin is composed of the 7-helical apoprotein opsin and a retinal chromophore, covalently attached by a protonated Schiff base. Light causes retinal isomerization which in turn triggers conformational changes of opsin then forming the ion conductive pore. First information on the channelrhodopsin photocycle came from electrical measurements and from time-resolved UV-visible spectroscopy (Stehfest and Hegemann, 2010). Further structural information was revealed by the X-ray structure (Kato et al., 2012). However, still to date a little is known about its exact gating mechanisms and photocycles.

Providing information on a molecular level, infrared (IR) spectroscopy has become an important tool for investigation of structure/function relationships in proteins. An overview of its applications in biophysics is given in (Siebert and Hildebrandt, 2008). The most commonly used spectral region is between ~800 and ~2500 cm^−1^ (4–12.5 μm) (Barth, 2007) and a resolution better than 8 cm^−1^ is usually desired. One advantage over other commonly used methods, EPR, NMR, or X-ray crystallography for instance, is that IR investigates systems in their native environment. However, a drawback of this technique is that the extinction coefficients of most functional groups are low (see Barth, 2007). To compare, in the UV-visible region, the protonated Schiff base absorbs near 500 nm with an extinction coefficient of ~40,000 M^−1^ cm^−1^ (Bridges, 1971) whereas in the IR-spectrum, the Schiff base protonation can be indirectly assigned by the strong protonated carboxylate C=O stretching mode of the corresponding counter ions. For a glutamate or aspartate, the extinction coefficient (~200–300 M^−1^ cm^−1^) is over 100 times lower. This mini-review focuses on current IR-spectroscopic techniques and their applications to the study of proteins like channelrhodopsin.

## IR-spectroscopy of channelrhodopsin

**Figure 1:**
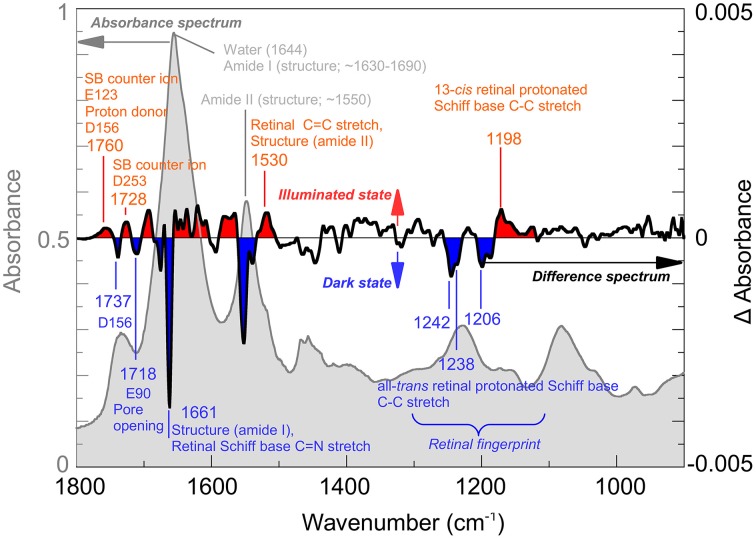
**Infrared spectroscopy of Channelrhodopsin**. The absorption spectrum (*gray*) of retinal proteins like Channelrhodopsin-2 reconstituted in lipid vesicles shows bands associated with the lipid environment and protonated carboxyl groups (~1700–1800 cm^−1^), water (1644 cm^−1^) and the overall helical structure of the protein (amide I ~1650 cm^−1^; amide II ~1550 cm^−1^). Note, that the lipid vesicles allow a very dense packing of the protein in the cuvette thus reducing the water content. Light induced alterations are represented by the difference spectrum (*black*), where negative bands (*blue*) occur due to the dark state while positive bands (*red*) are due to the illuminated state, achieved by illumination with blue (480 nm) light. The spectrum was recorded at cryogenic conditions where a mixture of species, including the Schiff base deprotonated state and the conducting state is observed. Note that, while total absorbance is in the order of 0.9 OD (*left scale, gray*), largest changes in the difference spectrum are within 0.004 OD (*right scale, black*). In the picture, some bands assigned so far to their structural counterparts are marked. For details of the band assignments, see (Eisenhauer et al., 2012; Lórenz-Fonfría et al., 2013, 2015; Kuhne et al., 2015).

IR-spectroscopy was among the first techniques used to obtain structural information on the channelrhodopsin photocycle (Ritter et al., 2008; Radu et al., 2009). Since 2008, several bands have been assigned by biophysical methods such as site-directed mutagenesis, H_2_O/^2^H_2_O exchange or isotopic labeling. Figure 1 (*light gray*) shows the IR-spectrum of Channelrhodopsin-2 with some important bands marked. For example, its overall helical structure is typically discerned from the amide I and II bands (~1660 and ~1550 cm^−1^) (Bandekar and Krimm, 1979; Byler and Susi, 1986; Goormaghtigh, 1990). However, when only the modes that undergo a change during conformational alterations of the protein are of interest, the difference spectrum calculated by subtracting the spectrum of the functional (illuminated) state from the spectrum of the resting (dark) state is used (Figure 1*, black line*). Hereby structural changes connected with the pre-formation, opening or closing of the pore become visible. The band at 1661 cm^−1^ indicates conformational changes of the protein, the band pattern between 1100 and 1300 cm^−1^ reflects the all-*trans*/13-*cis* chromophore isomerization (Nack et al., 2009; Bruun et al., 2011), whereas changes in hydrogen bonding and proton transfers of functional aspartates and glutamates are seen between 1700 and 1800 cm^−1^. The protonation states and hydrogen bonding of the Schiff base counter ions E123 and D253 (1760 cm^−1^) and the proton donor D156 (1737 and 1760 cm^−1^) can be directly observed as well as the protonation state of E90 which, as a part of the central gate, plays a role in channel selectivity. For further band assignments see for instance (Kuhne et al., 2015; Lórenz-Fonfría et al., 2015) and citations therein.

The conductive state of channelrhodopsin arises within ~200 μs and decays within ~20 ms (Ernst et al., 2008). In contrast, the retinal isomerization occurs within femtoseconds (Neumann-Verhoefen et al., 2013), de- and re-protonation of the Schiff base is faster than 1 ms (Ernst et al., 2008), and the recovery of the fully dark-adapted state, thereby closing the photocycle, is accomplished within minutes (Ritter et al., 2008). In addition, multiple photocycles with different reaction kinetics exist in parallel (Hegemann et al., 2005), and depending on the illumination conditions, additional side-ways can be populated (Ritter et al., 2013). Therefore time-resolved methods covering time-regimes from femtoseconds to minutes are necessary to understand the structure-function relationships. In the following chapters, we review IR-spectroscopic methods with focus on temporal resolution, sample and technical requirements, as applied to the study of proteins like channelrhodopsin.

## Fourier-transform IR-spectroscopy

### Rapid-scan spectroscopy

**Figure 2:**
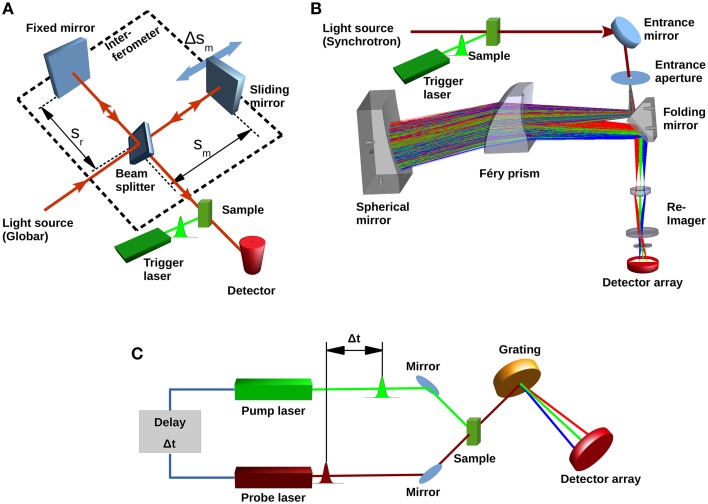
**Different types of IR spectrometers. (A)** Basic concept of a typical Fourier-transform infrared spectrometer showing light source (Globar), beam splitter, fixed and movable mirrors and single element infrared detector. Conformational changes in the sample are initiated by the trigger laser. The conversion of the sample can then be followed with a time resolution either determined by the sliding mirror movement (rapid-scan) or by the rise-time of the detector (step-scan). **(B)** Concept of a recently proposed dispersive device (Schade et al., 2014) with Synchrotron light source, dispersive prism and focal-plane array detector. **(C)** Laser based pump-probe setup. A first pulse from the pump laser starts the photoreaction. A subsequent short pulse from the probe laser probes the system. The probe pulse can be dispersed to obtain spectra; however, spectral bandwidth is determined by the duration of the probe pulse.

In Fourier-transform infrared (FTIR) spectrometers the light from a broadband IR-source passes an interferometer where the incident beam is split by a beam splitter. The partial beams are back-reflected to the beam splitter by two mirrors one of which is a sliding mirror introducing a position-dependent phase-shift. The beam splitter allows the partial transmission of the reflected beams to the detector, where an interference signal is recorded as a function of the optical path difference (Griffiths and De Haseth, 2007) (Figure 2A). This so-called interferogram is converted into a spectrum by a Fourier transformation (Herres and Gronholz, 1984). FTIR spectrometers benefit from high-throughput (Jacquinot), multiplex (Fellgett), and high registration precision (Connes) advantages (Perkins, 1987). The temporal resolution is only limited by the speed, sliding pathlength (corresponding to the resolution of the spectrum) and reversal-time of the movable mirror. For a spectrum of 4 cm^−1^ resolution, 40 ms time-resolution can be achieved (Smith and Palmer, 2002). Due to the symmetry of the interferogram around the position of equal optical path length (Δ*s* = 0), one movement of the mirror yields two spectra by splitting the interferogram at (Δ*s* = 0). Utilizing both forward- and backward movement of the mirror for data acquisition, a time-resolution of 10 ms is achieved. Further improvement to 5 ms (8 cm^−1^ resolution) was reported with the rapid-sweep method (Braiman et al., 1987). However, using sliding mechanisms means that after data acquisition the mirror has to be stopped and its direction reversed. This time-consuming process becomes significant when fast processes are investigated and the mirror is moved with high speed over a short distance. To avoid this, different types of interferometers have been utilized. For instance, a continuous rotary motion of a tilted mirror was used to measure an interferogram in less than 1 ms (4 cm^−1^ resolution) (Griffiths et al., 1999). However, difficulties in maintaining the alignment made an optical tilt-compensation necessary (Manning, 2002). Due to the limited time-resolution, rapid-scan FTIR is only suited to investigate the late stages of the channelrhodopsin photocycle. The conducting state can only be observed by this technique in exceptional cases, for example by cryotrapping or when slow-cycling mutants (i.e., ChR2-C128T, Berndt et al., 2009) are used (Stehfest et al., 2010).

### Step-scan spectroscopy

Here, time-courses at the particular interferogram data points corresponding to distinct mirror positions are recorded separately (Murphy et al., 1975). This is achieved by stopping the movable mirror, initiating the reaction to be followed and recording the time-trace while the mirror is at rest. The mirror is then moved to the next position (“step”). This process is repeated for each sampling point of the interferogram. Finally, the interferograms corresponding to given times after light flash are reconstructed using the intensities from the time-traces. This means that the experiment has to be repeated at least as often as the number of digital points of the interferogram which is usually more than 1000 times. The time-resolution is then only limited by the detector and the analog-digital converter of the acquisition system. Additional noise sources that potentially influence the experiments, for example instrument vibrations or slow source drifts are described by Andrews and Boxer (2001). To ensure sharp triggering (required for high time-resolution) and to minimize multi-photon processes, the reaction is triggered by a laser flash usually shorter than the desired time-resolution. Several techniques avoid the complicated process of stopping the mirror by utilizing the time delay between the subsequent digitized interferogram points. In these synchronized continuous-scan measurements, the experiment also has to be triggered for each interferogram data point (see Fleischmann et al., 2003 and citations therein).

Siebert and coworkers described the set-up of a step-scan device based on a commercial interferometer designed to study the photoreaction of bacteriorhodopsin with μs time resolution (Uhmann et al., 1991). With current set-ups, fast detectors and electronics, time-resolutions down to nanoseconds have been achieved (Garczarek and Gerwert, 2006). The step-scan technique is ideal for investigation of fast cycling non-degrading systems like bacteriorhodopsin, however its application to many other light-sensitive proteins can be difficult. For instance, the long recovery kinetics of most channelrhodopsins requires a prolonged delay between two subsequent light flashes. The recording time of a spectral data set with a resolution of 4–8 cm^−1^, a spectral width of ~1000 cm^−1^ and appropriate signal-to-noise ratio (~1000 experiment repetitions) can be in the order of days. For example, first results on channelrhodopsin activation, with 6 μs time-resolution took 5 days of accumulation time (Lórenz-Fonfría et al., 2013). Later the time-resolution was improved to the nanosecond range (Kuhne et al., 2015; Lórenz-Fonfría et al., 2015), however long measuring times are still an issue.

Non-cyclic systems can only be investigated using this technique when each point of the interferogram is recorded from a fresh sample. For liquid samples a flow-through cell is advantageous (Kaun et al., 2006), however for non-liquid ones, the sample has to be replaced once the time-course of a single data point of the interferogram has been measured. Set-ups utilizing rotating discs (Rödig and Siebert, 1999) or translational stages (Rammelsberg et al., 1999) have been developed for such cases. However, homogeneity of the samples is important here. For a more detailed review of the step-scan and other FTIR techniques, see (Kötting and Gerwert, 2005; Radu et al., 2011).

## Synchrotron based dispersive techniques

Dispersive spectrometer approaches have long been considered outdated since they typically suffer from low light intensity due to losses at the entrance slit and the dispersive grating and also from low data acquisition speed limited by the grating movement. Modern focal-plane-array (FPA) detectors allow simultaneous measurements of all data points. The light from the entrance slit, after passing through the grating, is imaged to the FPA where each detector element is used to record its own spectral interval.

To achieve sufficient spectral resolution, echelle gratings with higher diffraction orders are commonly used, particularly in astronomical sciences (Lacy et al., 1989). The low light intensity, and consequently the low signal-to-noise ratio makes them rather unsuitable for time-resolved IR-studies of proteins. Another drawback of gratings in combination with planar arrays is the significant curvature of the recorded spectral image (Pelletier et al., 2005), a problem which has to be addressed to avoid artifacts. Furthermore, array detectors require precise imaging of the entrance aperture at the detector elements and thus a highly brilliant light source such as that provided, for example by synchrotron radiation is particularly attractive. A conceptual design of a combined dispersive IR/X-ray spectroscopy set-up for simultaneous time resolved measurements using synchrotron light was proposed by (Marcelli et al., 2010). The high brilliance of the synchrotron IR-light allows optimal utilization of the spectrometer entrance aperture. Marcelli et al. calculated a signal-to-noise ratio of >1000 for integration times >0.3 μs using a time-resolved grating spectrometer in combination with a focal plane array and cooling all optical elements to 77 K.

A prism-based infrared spectrometer with synchrotron source, designed for single-shot measurements of photosensitive proteins like channelrhodopsin and enzyme rhodopsins is currently being developed (Schade et al., 2014). Design goals are microsecond time-resolution and a spectral resolution of 4–8 cm^−1^ in the 2000–950 cm^−1^ range while maintaining a signal-to-noise ratio of 1000 in single-shot mode. The concept is based on a Féry-spectrograph (Féry, 1911), where a prism consisting of two spherical surfaces is used. A spherical mirror behind the prism facilitates a second pass of the light (Figure 2B), and all spherical surfaces follow aplanatic conditions (Warren, 1997). This arrangement guarantees a coma and aberration free, non-tilted flat image of the entrance aperture in the image plane, and a high spectral resolution (Wilson, 1969). The usage of a prism rather than a grating has the advantage of higher optical transmission and the absence of interferences caused by order effects or stray light. The ray aberrations of this set-up were calculated to be less than 15 μm and therefore much smaller than the corresponding Airy disk, demonstrating the diffraction-limited operation over the whole spectral range. The expected signal-to-noise ratio calculation was based on parameters suitable for the IRIS Beamline at BESSY II (Peatman and Schade, 2001). For 1 μs accumulation time, a signal-to-noise ratio of ~600 was calculated for an operation temperature of 300 K, which improves to ~1000 when a cold-stop (77 K, f/1.5) in front of the detector array is introduced. This however requires a re-imaging system to map the image to the linear FPA through the cold-shield of the detector housing.

A direct comparison of the signal-to-noise ratio to other time-resolved methods like FTIR is rather complicated, since either the time-resolution is not achieved (rapid-scan methods are only applicable down to milliseconds), or the method is conceptually based on thousands of repetitions of the same experiment (step-scan). Using the data of (Schade et al., 2014) and neglecting other sources of noise in the setup, a signal-to-noise ratio of 10,000 is theoretically achievable by accumulating 100 measurements, corresponding to 100 μs accumulation time. This is comparable to the signal-to-noise ratio of rapid-scan FTIR experiments in the millisecond time range (for example-spectra of single-shot experiments, see Elgeti et al., 2008). The combination of synchrotron light with FPA detectors is largely compensating for the loss of FTIR advantages. This setup will allow for the direct observation of the formation and decay of the channelrhodopsin conductive state as well as crucial proton transfer reactions. For example, de- and re-protonation of the Schiff base, under native environmental conditions can be observed in single-shot mode thus avoiding possible sample degradation due to the long recovery period necessary for repetition-based methods like step-scan FTIR.

## Spectroscopy with lasers

Time-resolved infrared spectroscopy takes advantage of laser light sources. For example, a PbS diode laser has been used to record conformational changes of the Ras protein in the nanosecond time regime with a flash-photolysis set-up (Lin et al., 2014). The intensity of the laser beam was measured, after passing through the sample, by an infrared detector. In this case, the photoreaction was initiated by photolysis of caged compounds through a UV laser flash. Such setups however only allow the acquisition of signals at fixed wavelength. Quantum cascade lasers (QCLs) emitting in the mid- and far-infrared range are currently under heavy development. Their tunability and high output intensity, while maintaining a narrow bandwidth, make them ideal light sources for infrared spectroscopy. Intrinsic temperature fluctuations however introduce noise that has to be considered (Borri et al., 2011; Liu and Wang, 2011). Current developments in laser absorption spectroscopy based on QCLs are reviewed elsewhere (Zhang et al., 2014). They are becoming more frequently used in spectrochemical imaging (Clemens et al., 2014) and nanospectroscopy (Amenabar et al., 2013). A QCL-based spectrometer has been applied to study the first steps of the channelrhodopsin activation process (Lórenz-Fonfría et al., 2015). The authors used a tunable QCL in a flash-photolysis setup, where the laser is tuned to the desired wavelength, the photoreaction then initiated by a VIS flash and the time-dependent signal change recorded by an infrared detector. This procedure has to be repeated for each desired wavelength. A time-dependent dataset of channelrhodopsin in the range 1610 and 1680, and 1700 and 1780 cm^−1^ at a resolution of 1 and 0.5 cm^−1^ could thus be acquired with a repetition rate of 0.33 Hz by using a fast-cycling channelrhodopsin mutant (ChR2-E123T, Gunaydin et al., 2010).

For time-resolutions of nanoseconds or better, pump-probe technologies can be used. The photoreaction of a protein is started by a first laser pulse, usually in the fs time regime. A second pulse with a certain time delay probes the protein's response. For each pump-probe cycle, a difference spectrum can be obtained when the probe pulse, after passing the sample, is fed through a dispersive element and measured at an infrared detector array (Hamm and Zinth, 1995) (Figure 2C). An overview on how this is applied to dynamics of light-triggered proteins is given in Groot et al. (2007). This technique has been used to investigate ultrafast dynamics of bacteriorhodopsin, photoactive yellow protein (see for example, Van Wilderen et al., 2006), and LOV domains (Alexandre et al., 2009). Channelrhodopsin-2 was also studied by Vis-pump/IR-probe spectroscopy (Neumann et al., 2008) in the fs-timescale. The experiments showed amide-I vibrational modes occurring within ~500 fs thus demonstrating a very strong protein-chromophore coupling (Neumann-Verhoefen et al., 2013).

An alternative method to measure mid-infrared pulses is to optically convert them into the UV-visible range where a broad variety of array detectors is available. Zhu et al. (2012) used chirped pulse upconversion facilitated by a non-linear optical crystal. The authors investigated the photoreaction of BLUF photoreceptors on a picosecond time scale and demonstrated the method is suited for investigation of signal changes down to the mOD range.

## Summary/outlook

While the time regime of milliseconds and slower can be accessed by the rapid-scan FTIR technique for most biological samples, for faster systems special considerations have to be taken into account. Ultrafast alterations can be observed by pump-probe spectroscopy. Step-scan FTIR facilitates a good signal-to-noise ratio and a time-resolution down to nanoseconds but requires perfectly cyclic systems under investigations. For non-cyclic or slow cycling systems, fast time-resolved investigations are challenging. However, developments addressing this problem by QCL-based setups or dispersive spectroscopy in combination with highly brilliant light sources are in progress.

### Conflict of interest statement

The Guest Associate Editor, Tilo Mathes, declares that, despite having recently collaborated with the author PH, the review process was handled objectively. The authors declare that the research was conducted in the absence of any commercial or financial relationships that could be construed as a potential conflict of interest.
